# Immunogenicity and protective efficacy of a novel bacterium-like particle-based vaccine displaying canine distemper virus antigens in mice and dogs

**DOI:** 10.1128/spectrum.03477-23

**Published:** 2024-03-08

**Authors:** Jianzhong Wang, Lina Liu, Xianchun Zong, Chunliu Wang, Guangmei Zhu, Guilian Yang, Yanlong Jiang, Wentao Yang, Haibin Huang, Chunwei Shi, Yan Zeng, Nan Wang, Xin Cao, Chunfeng Wang, Na Feng

**Affiliations:** 1 College of Veterinary Medicine, Jilin Agricultural University, Changchun, China; 2 Jilin Provincial Engineering Research Center of Animal Probiotics, Jilin Provincial Key Laboratory of Animal Microecology and Healthy Breeding, Jilin Agricultural University, Changchun, China; 3 Engineering Research Center of Microecological Vaccines (Drugs) for Major Animal Diseases, Ministry of Education, Jilin Agricultural University, Changchun, China; 4 Changchun Veterinary Research Institute, Chinese Academy of Agricultural Sciences, Changchun, China; 5 College of Veterinary Medicine, Jilin University, Changchun, China; Shandong First Medical University, Jinan, China

**Keywords:** bacterium-like particles, canine distemper virus, subunit vaccine, surface display technology, immune response

## Abstract

Canine distemper virus (CDV) poses a severe threat to both domesticated and wild animals, including multiple carnivores. With the continued expansion of its host range, there is an urgent need for the development of a safer and more effective vaccine. In this study, we developed subunit vaccines based on a bacterium-like particle (BLP) delivery platform containing BLPs-F and BLPs-H, which display the CDV F and H glycoprotein antigens, respectively, using the antigen-protein anchor fusions produced by a recombinant baculovirus insect cell expression system. The combination of BLPs-F and BLPs-H (CDV-BLPs), formulated with colloidal manganese salt [Mn jelly (MnJ)] adjuvant, triggered robust CDV-specific antibody responses and a substantial increase in the number of interferon gamma (IFN-γ)-secreting CD4^+^ and CD8^+^ T cells in mice. Dogs immunized intramuscularly with this vaccine not only produced CDV-specific IgG but also displayed elevated concentrations of IFN-γ and interleukin 6 in their serum, along with an increase of the CD3^+^CD4^+^ and CD3^+^CD8^+^ T cell subsets. Consequently, this heightened immune response provided effective protection against disease development and reduced viral shedding levels following challenge with a virulent strain. These findings suggest that this BLP-based subunit vaccine has the potential to become a novel canine distemper vaccine.

**IMPORTANCE:**

Many sensitive species require a safe and effective distemper vaccine. Non-replicating vaccines are preferred. We constructed subunit particles displaying canine distemper virus (CDV) antigens based on a bacterium-like particle (BLP) delivery platform. The CDV-BLPs formulated with theMn jelly adjuvant induced robust humoral and cell-mediated immune responses to CDV in mice and dogs, thereby providing effective protection against a virulent virus challenge. This work is an important step in developing a CDV subunit vaccine.

## INTRODUCTION

Canine distemper (CD), caused by the canine distemper virus (CDV), is a multisystemic infectious disease with a high morbidity and mortality rate in a wide range of wild and domestic carnivorous hosts. Although CDV was originally described as a pathogen of domestic dogs, its ability to adapt to new hosts has led to its involvement in a diverse array of host species besides dogs, including canids (such as silver foxes, Ethiopian wolves, jackals, dingoes, and coyotes), felids (such as lions, tigers, and leopards), cercopithecoids (rhesus monkey, cynomolgus macaques, and Japanese monkeys), procyonids (raccoons), ursids (such as giant pandas and bears), mustelids (ferrets and minks), hyenids, and others (1). Although domestic dogs serve as the primary hosts for CDV, they pose a significant threat to wildlife populations due to virus transmission. The potential for CDV spillover from urban wildlife to domestic dogs also exists (2). A meta-analysis revealed a CDV prevalence of 22% in domestic dogs in China (3). Additionally, CDV outbreaks in fur-bearing animals (minks, foxes, and raccoon dogs) have been frequently reported in China’s Shandong province and northeast region since 2012 (4, 5). Therefore, CDV continues to circulate among multiple host species, emphasizing the need for precautionary measures.

CDV is an enveloped non-segmented negative-strand RNA virus that belongs to the genus *Morbillivirus* within the family Paramyxoviridae (6). The viral envelope consists of a double lipid layer derived from the host cell membrane, incorporating two viral glycoproteins: hemagglutinin (H) and the fusion protein (F). Beneath the viral envelope membrane, matrix (M) proteins maintain the viral morphology and structure, bridging the viral glycoproteins and the ribonucleoprotein complex (RNP) of paramyxoviruses, which comprise the nucleocapsid protein (N), phosphoprotein (P), large protein (L), and the viral RNA (7). Although only one serotype of CDV has been recognized, there are at least 18 major genetic lineages/genotypes co-circulating, based on the H-gene, which displays the highest sequence variability among CDV genes, including North America 1–5, South America 1/Europe 1, South America 2–4, European Wildlife, Arctic-like, Rockborn-like, Africa 1–2, Asia 1–4, and other newly identified genotypes (8–10). The F gene is the second most variable segment within the CDV genome (11). Both the H and F proteins play a crucial role in viral attachment, membrane fusion, and initiation of specific cellular and humoral immune responses against CDV (12). Previously, several studies have indicated that the CDV H and F proteins are promising candidate antigens for the design of DNA vaccines (13–15), subunit vaccines (16, 17), epitope-based vaccines (18, 19), and vectored vaccines (20–22).

Vaccination is the most effective method for preventing viral infections and their transmission by stimulating both humoral and cellular responses through B and T lymphocytes. The majority of commercially available vaccines are live modified vaccines based on strains such as Onderstepoort, Snyder Hill, Convac, Rockborn, or CDV3. However, these live-attenuated vaccines can induce symptomatic diseases and even cause mortality in certain susceptible species, as they retain their ability to replicate in vaccinated animals (23). Vaccinating young animals with maternal CDV-neutralizing antibodies, which can interfere with the effectiveness of an attenuated live CDV vaccine, would be inefficient. While subunit or novel epitope vaccines were capable of eliciting high levels of immune response in mice, there remains a lack of data confirming their ability to provide adequate immune protection against distemper for natural hosts. Consequently, there is an urgent necessity to develop alternative types of CD vaccines.

Bacterium-like particles (BLPs), derived from food-grade lactic acid bacteria, are spherical peptidoglycan microparticles that mimic the shape, size, and surface properties of live bacteria. However, they are non-living and non-infectious (24). Due to their capacity to resemble live bacteria and induce an immune response, BLPs could serve as a vaccine platform for delivering antigens fused to an anchor domain that binds to the peptidoglycan skeleton in a non-covalent manner (25). Typically, the protein anchor (PA) is derived from the cell wall peptidoglycan binding domain found in the C-terminal part of the lactococcal cell wall hydrolase AcmA. Since the antigen-PA fusions are produced in systems like *Escherichia coli* or eukaryotic expression systems rather than lactic acid bacteria, this approach can be referred to as heterologous surface display (26). Through simple centrifugation, the cell-free culture medium can be used directly as the source of the chimeric fusion protein for BLP-based vaccines. This streamlined procedure circumvents the need for time-consuming and intricate purification steps. This process eliminates the requirement for time-consuming and intricate purification steps, potentially making it cost effective and preferable for industrial production (27). Furthermore, immunogen molecules are intricately packed as high-density nanoclusters on the BLP particle (28). In addition to carrying specific antigens loaded on the surface of BLPs to activate adaptive responses, BLPs, which act as TLR2 agonists, have the ability to initiate the innate immune pathways by interacting with Toll-like receptors (29, 30). Consequently, BLPs provide an ideal platform for antigen delivery.

To develop a subunit vaccine based on BLPs-PA platform for CD, in this study, we constructed recombinant baculoviruses expressing recombinant F or H antigens fused with PA considering that the envelope glycoproteins are important antigenic proteins. The production of expression was collected and subsequently loaded onto the BLPs, respectively. Mice and dogs were immunized with the BLP-based CDV vaccine in combination with Mn jelly (MnJ) adjuvant, resulting in a significant humoral and cellular-mediated immune response. This formulation was effective in protecting immunized animals from developing the disease and reducing viral shedding after challenging with a virulent strain.

## RESULTS

### Construction and expression of the F-PA and H-PA fusion protein

**Fig 1:**
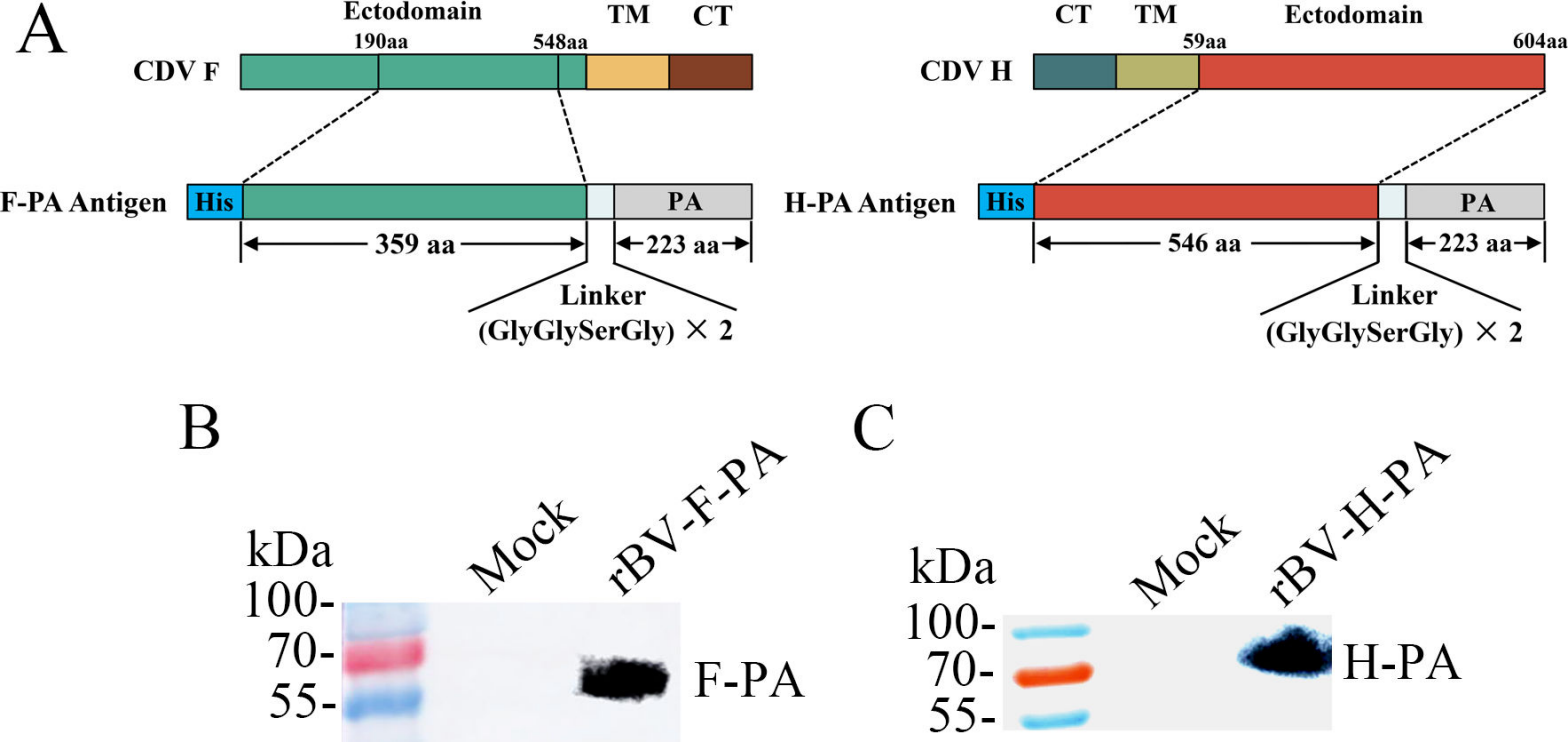
Expression of the fusion proteins F-PA and H-PA using the baculovirus expression system. (**A**) Schematic representation of the design of the recombinant F-PA and H-PA antigens. TM, transmembrane domain; CT, cytoplasmic domain. (**B, C**) Detection of the F-PA and H-PA proteins expressed in baculovirus-infected Sf9 cells. The supernatant from Sf9 cells infected with the recombinant baculovirus rBV-F-PA or rBV-H-PA was subjected to western blot analysis. Uninfected Sf9 cells were used as a control (Mock).

Using the baculovirus expression system, we constructed recombinant baculoviruses, namely, rBV-F-PA and rBV-H-PA, which expressed the F and H antigen, and fused the PA with a linker, as illustrated in (Fig. 1A). The expression of fusion proteins was confirmed by western blot analysis of the supernatant from Sf9 cells infected with the rescued recombinant baculoviruses using His Tag or FLAG Tag monoclonal antibodies. These results clearly demonstrated that the recombinant F-PA and H-PA antigens were successfully expressed and secreted into the supernatant (Fig. 1B and C).

### Display of the fusion proteins on BLPs

**Fig 2:**
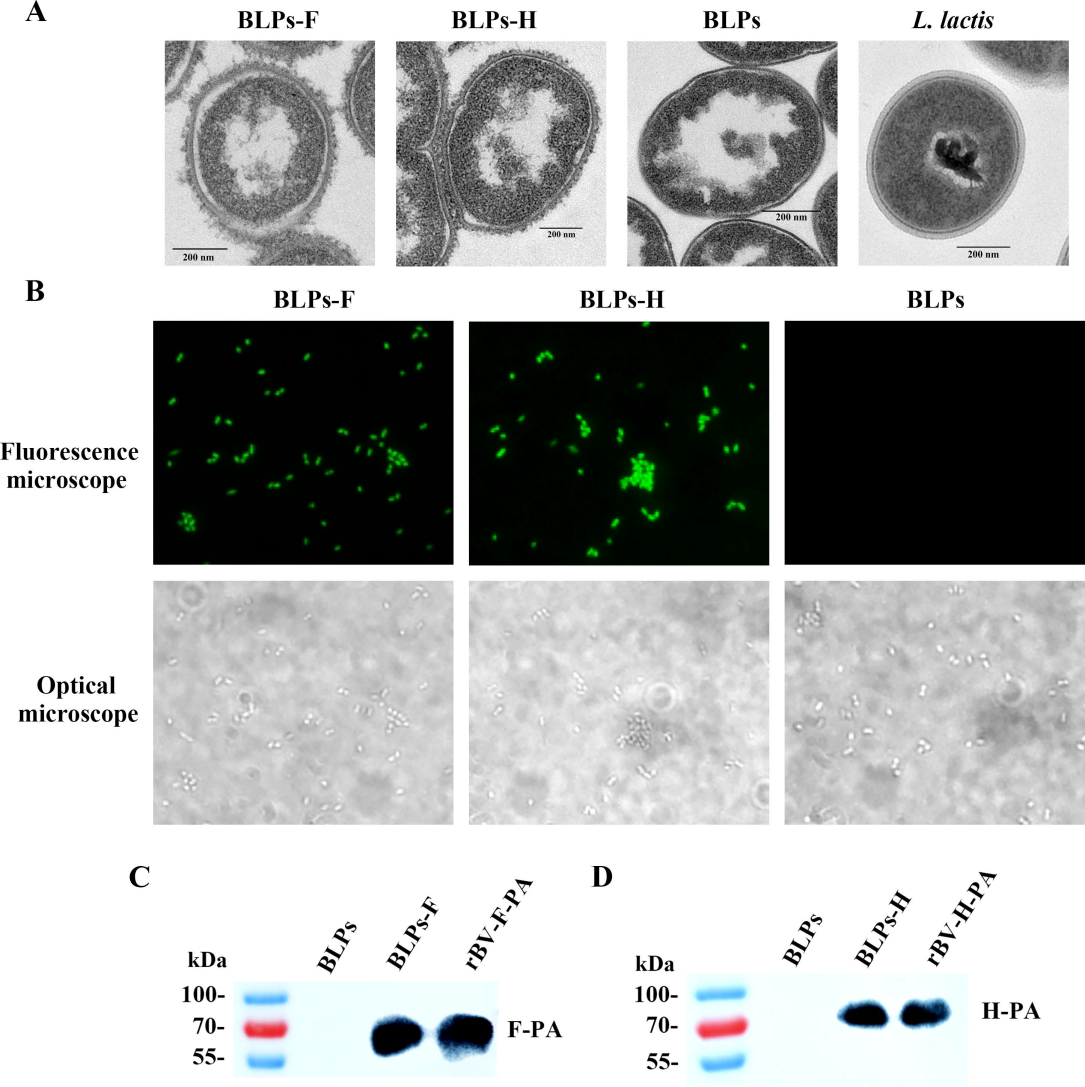
Analysis of fusion protein displayed on the surface of BLPs. (**A**) Electron microscopy observation of BLPs-F and BLPs-H. Naked BLPs and *L. lactis* were used as controls. (**B**) IFA analysis of the loading of the F-PA and H-PA fusion proteins onto BLPs using His Tag antibodies (microscopy images magnified at 1,000×). (**C, D**) Western blot analysis of the binding of F-PA and H-PA fusion protein to BLPs.

As shown in Fig. 2, BLPs maintained the original morphology and size of the *Lactococcus lactis* skeleton with a smooth surface and hollow interior. After incubation with culture supernatants containing recombinant F-PA or H-PA proteins, numerous floccules were observed on the surface of BLPs-F or BLPs-H, respectively, indicating the successful anchoring of the F-PA or H-PA proteins (Fig. 2A). The F or H antigens displayed on the BLPs were recognized by His Tag antibodies, as confirmed by immunofluorescence assay (IFA), whereas no signal was detected for the naked BLPs (Fig. 2B). Furthermore, western blot results also confirmed the loading of F-PA and H-PA fusion proteins onto BLPs, respectively (Fig. 2C and D).

### Immunogenicity evaluation in mice

**Fig 3:**
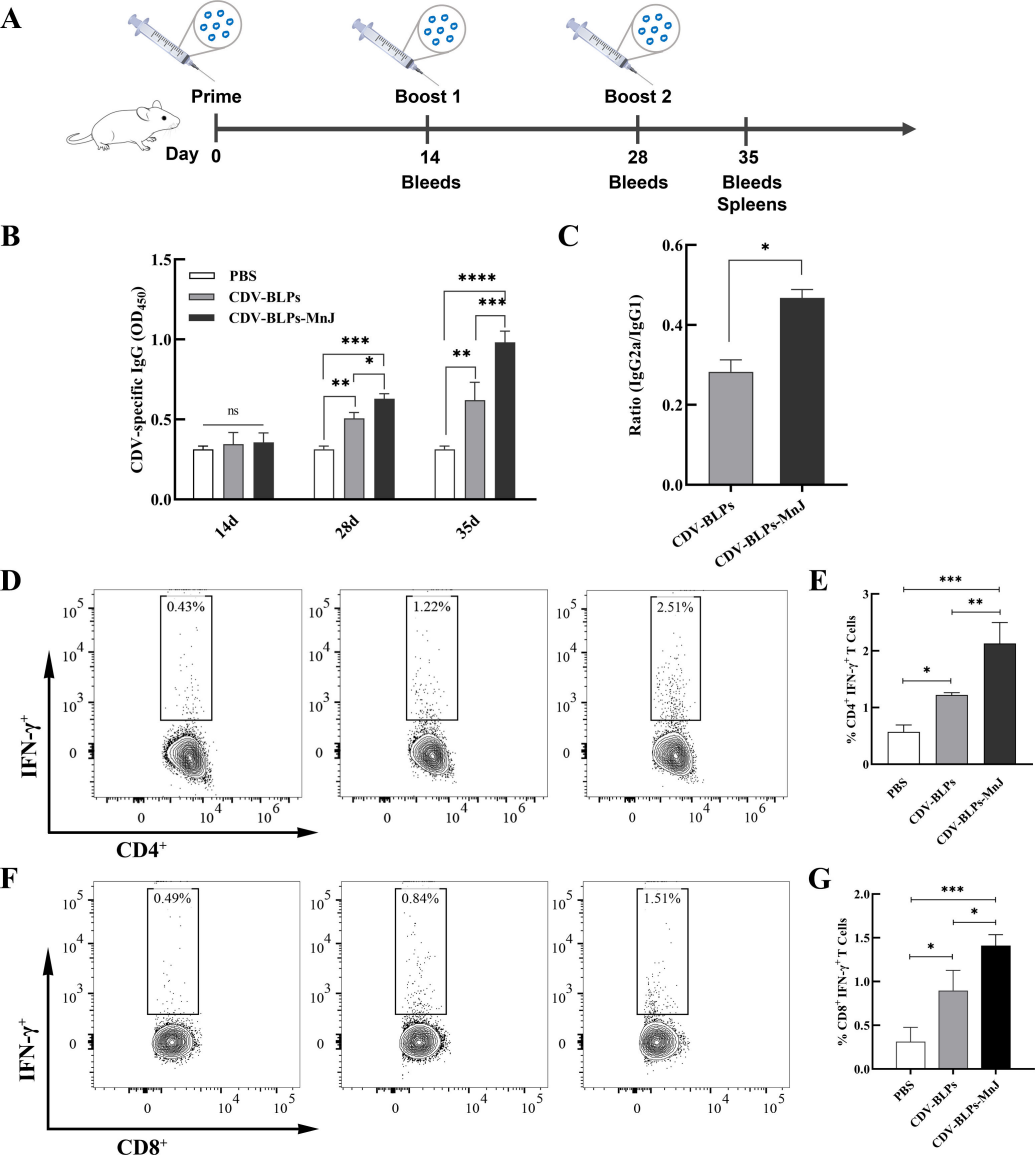
Characterization of the immune response induced by CDV-BLP-based vaccines in BALB/c mice. (**A**) Mouse immunization schedule. (**B**) Detection of CDV-specific antibodies. Serum samples were collected at 14, 28, and 35 days after primary immunization, followed by the detection of antigen-specific IgG using ELISA. (**C**) Ratios of IgG2a/IgG1 were determined from quantitative measurements of CDV-specific serum IgG2a and IgG1. (**D, F**) Analysis of IFN-γ expression in CD4^+^ T cells and CD8^+^ T cells, respectively, from mouse spleens using flow cytometry after the final immunization. (**E, G**) Frequency of IFN-γ-producing CD4^+^ T cells and IFN-γ-producing CD8^+^ T cells in mice was calculated. Data are expressed as the mean ± standard deviation (SD) of three independent experiments (*P < 0.05; **P < 0.01, and ***P < 0.001).

To evaluate the immunogenicity of CDV-BLPs and MnJ-adjuvanted CDV-BLPs, mice were inoculated with CDV-BLPs or CDV-BLPs-MnJ vaccine. Serum samples were collected from the mice after vaccination and subjected to enzyme-linked immunosorbent assay (ELISA) to determine the IgG titer. As shown in Fig. 3B, both CDV-BLPs and CDV-BLPs-MnJ vaccines elicited robust CDV-specific antibody responses after the booster in mice. Furthermore, the levels of IgG in the CDV-BLPs-MnJ group were significantly higher than those in the CDV-BLPs group. To assess the type of immune response, the ratio of IgG2a to IgG1 in serum collected 35 days post-immunization was calculated by dividing absorbance values obtained in IgG1 and IgG2a ELISA. The mean ratios of IgG2a to IgG1 were below 0.5 in the sera drawn from both the CDV-BLPs and CDV-BLPs-MnJ groups, indicating a Th2-oriented response. However, the MnJ-adjuvanted vaccine stimulated higher ratios of IgG2a/IgG1, suggesting a mixed Th1/Th2 response skewed toward a Th1 type (Fig. 3C). To evaluate the cell-mediated immune responses, interferon gamma (IFN-γ)-producing CD4^+^ and CD8^+^ T-cell responses in the spleens were analyzed by flow cytometry. The immunized group showed a high number of CDV-specific IFN-γ-secreting CD4^+^ and CD8^+^ T cells compared to the control group. Additionally, the frequencies of CD4^+^IFN-γ^+^ and CD8^+^IFN-γ^+^ T cells in the spleen were significantly higher in the CDV-BLPs-MnJ group than in the CDV-BLPs group (Fig. 3E and G).

### Immune response and protection against CDV in dogs

**Fig 4:**
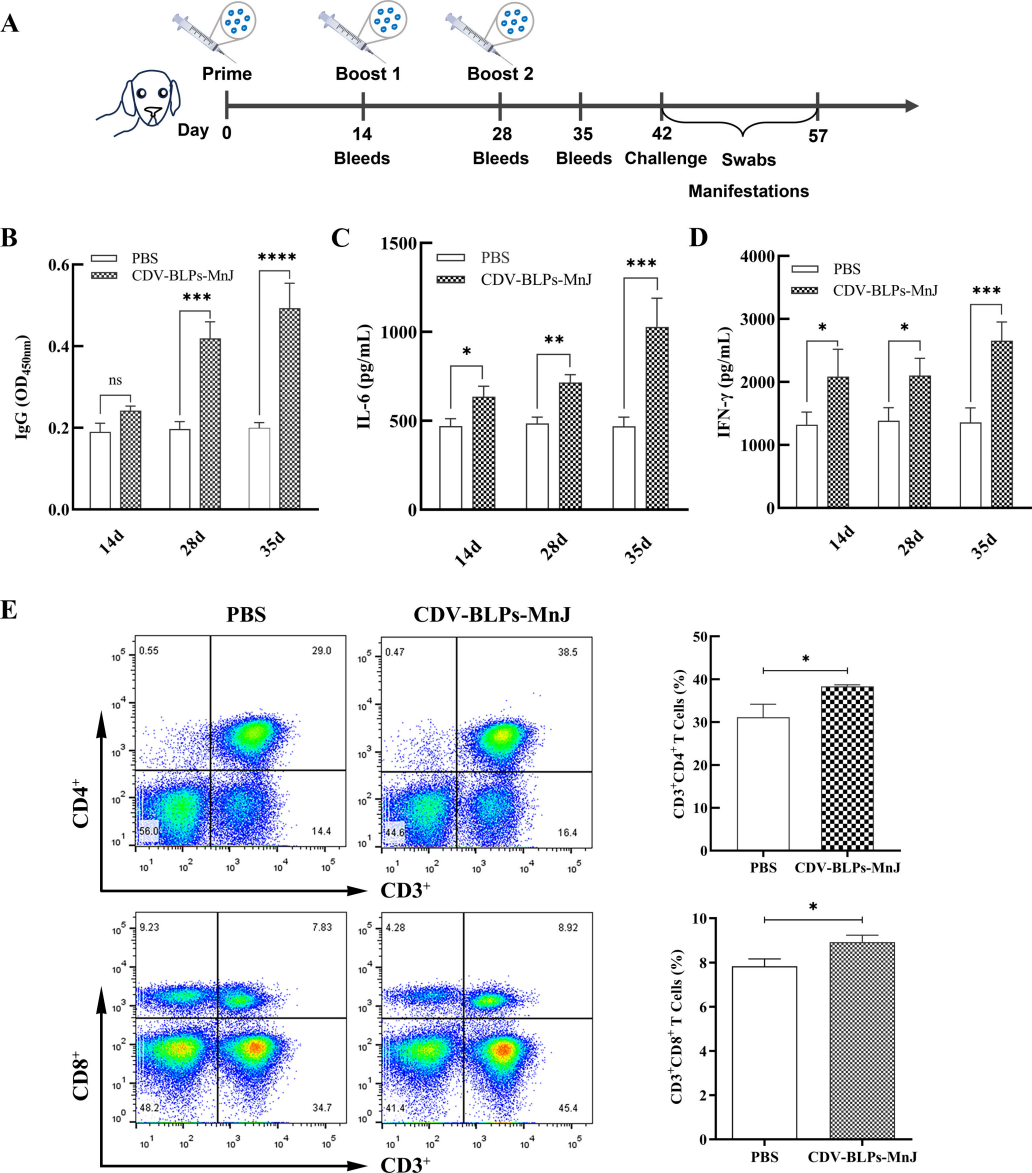
Characterization of the immune response induced by CDV-BLP-based vaccines in beagle dogs. (**A**) An illustration of the dog experiment is shown. Dogs were immunized with MnJ-adjuvanted CDV-BLP vaccine. Blood samples were collected at 14, 28, and 35 days after the primary immunization. On day 42 post-immunization, the dogs were challenged with the virulent CDV strain. (**B**) CDV-specific IgG was detected using ELISA. (**C, D**)The concentrations of IL-6 and IFN-γ in the serum were measured using commercial ELISA kits. (**E**) After the third immunization, PBLs were isolated and stained with a dog T Lymphocyte Cocktail. The percentages of CD3^+^CD4^+^ and CD3^+^CD8^+^ T cells were analyzed by flow cytometry. Data are expressed as the mean ± SD. *P < 0.05; **P < 0.01, and ***P < 0.001.

Dogs were immunized with CDV-BLPs-MnJ or phosphate-buffered saline (PBS). Serum samples collected at 14, 28, and 35 days were subjected to IgG, interleukin 6 (IL-6), and IFN-γ measurements, respectively. As shown in Fig. 4B, following the initial immunization, a slight increase in CDV-specific antibodies was detected in the CDV-BLPs-MnJ group, but the difference compared with the PBS group was not significant. However, after the booster immunization, the antibody levels significantly increased, indicating a robust immunological response. The concentrations of IL-6 and IFN-γ in the serum were significantly elevated (*P*  <  0.05) in dogs vaccinated with CDV-BLPs-MnJ compared with the PBS group (Fig. 4C and D). To assess T cell responses, peripheral blood lymphocytes (PBLs) were isolated and subjected to flow cytometric analysis after the final immunization. The analysis revealed a significant increase in the population of CD3^+^CD4^+^ and CD3^+^CD8^+^ T cell subsets in CDV-BLPs-MnJ-immunized dogs compared with the PBS group (Fig. 4E).

### Protection efficiency against CDV challenge in dogs

**Fig 5:**
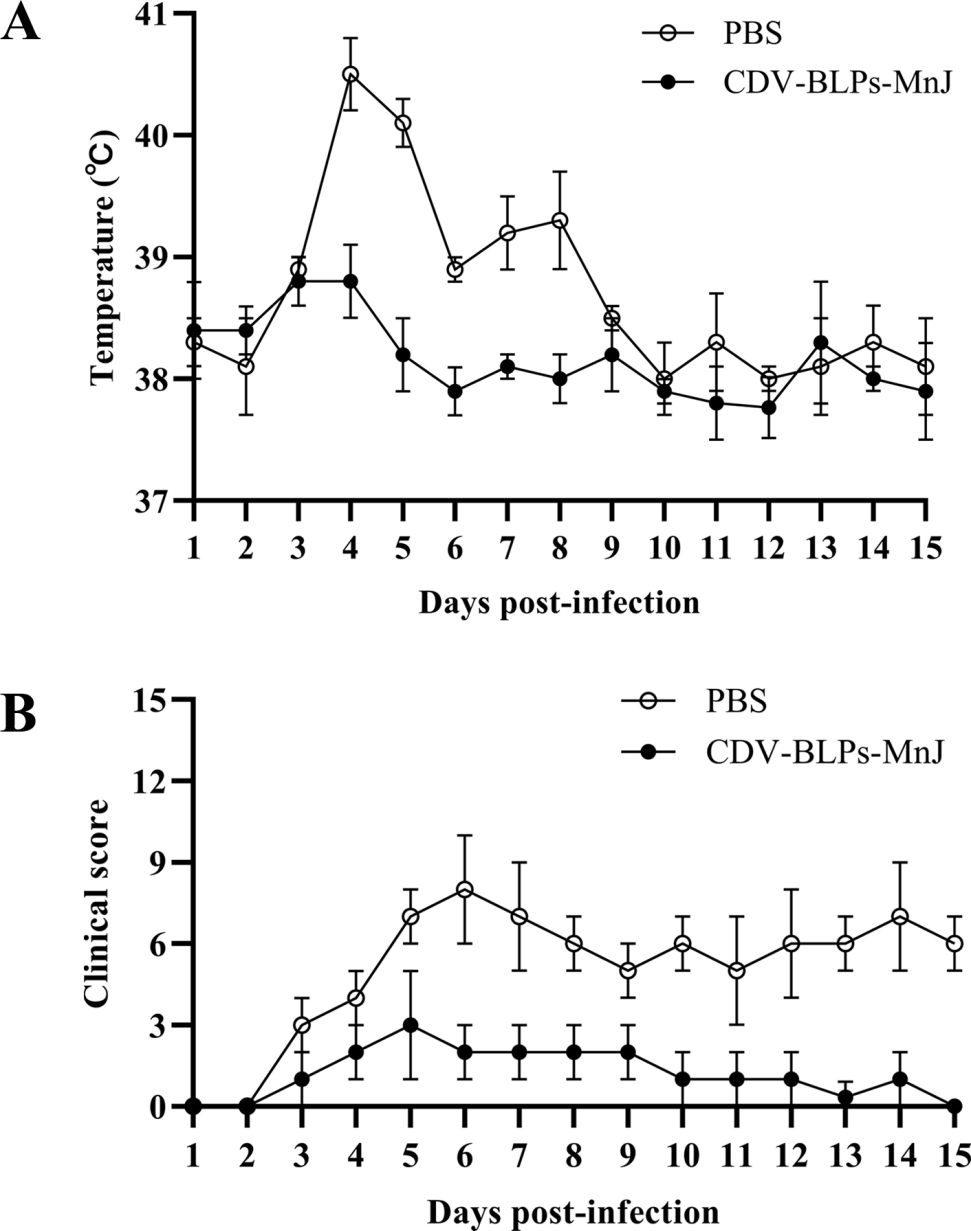
Clinical manifestations after challenge. (**A**) Body temperature was measured after challenge with the virulent CDV strain. (**B**) The total clinical score for each dog was calculated as the sum of the scores for the five signs. The clinical score is expressed as the mean ± SD.

**Fig 6:**
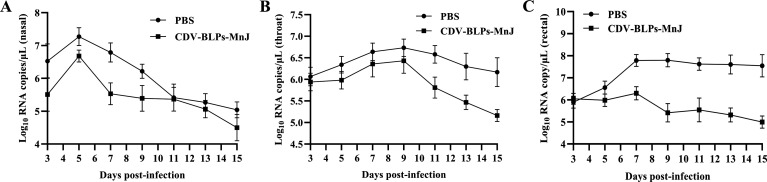
Viral shedding in dogs after challenge. Quantification of CDV RNA was performed by real-time quantitative in nasal (**A**), throat (**B**), and rectal (**C**) swabs at indicated time points. Results are presented as the mean copy number ± SD.

To evaluate the protection conferred by CDV-BLPs-MnJ, the dogs were challenged with virulent CDV after the third immunization. Dogs in the PBS group developed fever with a biphasic thermal response, a characteristic sign of canine distemper, while the dogs that received CDV-BLPs-MnJ experienced a slight increase in body temperature during the early stages of infection, which quickly returned to normal (Fig. 5A). According to the scoring system that assessed general condition, appetite, fecal consistency, ocular discharge, and nasal discharge, CDV-BLPs-MnJ effectively protected animals from developing severe canine distemper disease (Fig. 5B). CDV RNA was detected in nasal, throat, and rectal swabs as early as 3 days post-challenge. As shown in Fig. 6, CDV-BLPs-MnJ reduced the overall viral shedding among vaccinated dogs, while the PBS group exhibited a high level of viral shedding (Fig. 6).

## DISCUSSION

The broad and expanding host range of CDV poses a significant challenge for controlling and eradicating this disease. CDV can have severe impacts on many endangered species, including giant pandas. We previously reported the death of five captive giant pandas due to CDV infection (31). Therefore, it is advisable to administer vaccines to non-domestic animals in zoo collections. Due to its proven safety, the canarypox-vectored CDV vaccine was recommended for all susceptible species by the American Association of Zoo Veterinarians’ Distemper Vaccine subcommittee (23). However, this vaccine is not available in several countries. Furthermore, there exists notable individual variation among animals, as well as variation across species, in response to this vaccine. Previously, we enhanced this vaccine by incorporating the M gene expression cassette, leading to the successful production of CDV virus-like particles within infected cells (32). However, the production of canarypox-vectored vaccines still relies on primary chicken embryo fibroblasts, which presents limitations including high cost, finite *in vitro* life span, as well as laborious and tedious preparation. In this study, we utilized the BLPs-PA antigen delivery system to develop a novel subunit vaccine that is safe, cheap, easy to prepare, and suitable for mass production.

Based on the characteristics of the displayed immunogen, either a prokaryotic expression system or a eukaryotic expression system was used to construct the BLP-based vaccine. Since the immunoprotective antigens of CDV are the F and H envelope glycoproteins, the insect cell expression system was employed to produce recombinant glycoproteins-PA, similar to the approach we used for developing other BLP-based vaccines that display viral glycoprotein antigens, particularly with Newcastle disease virus (NDV) (33–35). The expression of the fusion protein in a soluble secreted form offers significant advantages for large-scale preparation of BLP-based vaccines. This is evident in our procedure where the supernatants from infected sf9 cells containing the antigen-PA fusion proteins can be directly mixed with naked BLPs for binding. However, achieving secretion of CDV glycoproteins-PA proteins proved challenging even when utilizing various insect secretion signal peptides. Despite achieving secretion via the GP64 signal peptide, the secretion level is still not as high as that of NDV glycoprotein-PA antigens, which also belong to the paramyxovirus family (data not shown).

Previous studies have demonstrated that the BLP vector exhibited immunomodulatory properties. Consequently, in the absence of any additional adjuvants, BLP-based vaccines induced high levels of humoral, cellular, and mucosal immune responses (36–38). However, the adjuvant effect of BLPs depends on the route of administration (39). To enhance the immunogenicity of intramuscular BLP-based vaccines, formulations were prepared with AddaVax, ISA 201VG, and/or Poly (I:C) adjuvants (33, 40, 41). In this study, CDV-BLPs adjuvanted with MnJ demonstrated robust immunogenicity in terms of both antibody and T cell responses in mice and dogs, aligning with findings from previous studies. Manganese is one of the most abundant metals in the tissues of mammals and is required for a variety of physiological processes, including its newly discovered immunomodulatory function (42). Mn_2_^+^ strongly promotes immune responses by facilitating antigen uptake, presentation, and germinal center formation via both cGAS-STING and NLRP3 activation (43). Wang et al. have shown that MnJ, serving as a novel adjuvant for a rabies vaccine, can boost the immune response in mice, cats, and dogs (44). This finding inspired us to choose MnJ as the adjuvant for CDV-BLPs.

In humans, virus-neutralizing antibodies are the gold standard for determining vaccine efficacy. However, in the mouse vaccine assessment model, detecting IgG, IgG1, and IgG2a antibody titers and isotypes measured by ELISA provides a better correlation with vaccine efficacy than neutralization alone (45). In mice, T helper (Th) 1-dependent IFN-γ induces the production of IgG2a, whereas the Th2 cytokine IL-4 stimulates the expression of IgG1, rendering each isotype an indicator of the underlying Th cell response (46). Therefore, the parameters of IgG2a/IgG1 ratios were measured in this study as Th1/Th2 deviation markers to characterize the vaccine immune response.

Mice inoculated with the CDV-BLPs vaccine exhibited an IgG2a/IgG1 ratio of less than 0.5, indicating a mixed Th1/Th2 response skewed toward Th2 (Fig. 3B). This trend is consistent with previous studies on BLP-vectored vaccines (40, 47). It is worth noting that the IgG2a/IgG1 ratio increased in mice that received MnJ-supplemented CDV-BLPs. Previous studies have shown that MnJ facilitates a Th1-biased immune response for inactivated rabies vaccine and ovalbumin antigen (43, 44). Our results also indicated that the MnJ adjuvant shifts CDV-BLP-induced immunity from a Th2-oriented response toward a Th1-type response, resulting in a more balanced Th1 and Th2 response.

Cellular immunity, mediated by both CD4^+^ and CD8^+^ T cells, plays an essential role in protecting against infectious diseases and their outcomes. Indeed, a morbillivirus infection-induced T cell response alone is sufficient for protection from reinfection (12). It has been reported that CDV H is a target for cytotoxic T cells (48), and a T-helper cell epitope has been defined on the F protein of CDV (49). IFN-γ is a key cytokine in cell-mediated immunity that is produced mainly by effector CD8^+^ T cells and Th1 CD4^+^ T cells (50). To evaluate cell-mediated immune responses, IFN-γ-producing CD4^+^ and CD8^+^ T cell responses were analyzed in mouse spleen lymphocytes using flow cytometry. Significant numbers of CDV-specific IFN-γ-secreting CD4^+^ and CD8^+^ T cells were observed in the immunized mice. Moreover, the MnJ adjuvant further enhanced both of these responses (Fig. 3). The same trend was observed in dogs, where the MnJ-adjuvanted CDV-BLP vaccine significantly increased the number of CD3^+^CD4^+^ and CD3^+^CD8^+^ T-cell subsets (Fig. 4). These data indicate that the CDV-BLP vaccine can induce both humoral and cellular immune responses.

To evaluate the protective efficacy of the CDV-BLPs-MnJ as a new candidate vaccine against CDV infection, the dogs were challenged with virulent CDV TM-CC strain isolated from dogs after the third immunization. Dogs in the PBS group developed a high body temperature with a biphasic thermal response and exhibited typical clinical symptoms of CDV infection. In contrast, dogs immunized with CDV-BLPs-MnJ experienced only a slight increase in body temperature during the early stages of infection and showed very mild clinical changes. Regarding viral shedding, CDV RNA was detected by real-time quantitative (RT-qPCR) in nasal, throat, and rectal swabs from 3 days post-infection. It was found that although the immunized group also exhibited viral shedding throughout the entire testing period, the levels were much lower compared with the control group. A similar phenomenon was observed in a previous study by Jiang et al., where a probiotic vaccine expressing the CDV H protein provided complete protection against CDV upon intranasal inoculation but did not completely stop virus shedding (51). Unfortunately, no fatalities occurred in the PBS group after challenging in our study, making it difficult to determine the full extent of the immune-protective effect of the CDV-BLPs-MnJ vaccine. This may be due to an insufficient challenge dose of CDV virulent strain. In our future studies, we plan to use a higher challenge dosage to evaluate the true immune protection efficacy of CDV-BLPs-MnJ. Additionally, it is worth noting that a tetrameric H glycoprotein and a trimeric F glycoprotein naturally exist on the surface of CDV particles (7). We plan to further enhance the immunogenicity of CDV-BLPs by expressing the CDV antigen in a trimer form, as has recently been demonstrated with BLPs displaying HIV trimeric antigens (52, 53) or the receptor-binding domain (RBD) of severe acute respiratory syndrome coronavirus 2 (41). These studies have shown promising results in terms of immune protection, as envelope glycoprotein trimers have appeared to be more effective than their monomeric counterparts in eliciting protection (54–56).

In conclusion, we have successfully constructed BLPs-F and BLPs-H, which display the CDV F and H glycoprotein antigens, respectively, using the antigen-PA fusion approach produced by the baculovirus insect cell expression system. The mixture of BLPs-F and BLPs-H (CDV-BLPs) induced robust and specific immune responses in mice, with and without the MnJ adjuvant. The MnJ-adjuvanted CDV-BLPs stimulated a strong humoral and cellular-mediated immune response in dogs, effectively protecting immunized animals from developing the disease and reducing shedding levels after challenging with a virulent strain.

## MATERIALS AND METHODS

### Bacterial strains, cells, and virus

The *L. lactis* MG1363 strain was grown at 30°C in M17 broth (Oxoid Ltd., Basingstoke, UK) supplemented with 0.5% glucose (GM17). Sf9 insect cells were maintained in SF900II (Life Technologies, San Diego, CA, USA) at 27°C, and suspension cultures were kept at a 27°C incubator while shaking at 120 rpm. A live canine distemper vaccine (CDV-11 strain) was purchased from Qilu Animal Health Products Co., Ltd. (Jinan, China).

### Construction and expression of recombinant baculoviruses

The genes coding for ectodomains of F (amino acids 190–548) and H (amino acids 59–604) of the CDV-11 strain were individually amplified by RT-PCR using the oligonucleotide primers CDV-F-F/CDV-F-R and CDV-H-F/CDV-H-R, with viral RNA as the template (Table 1). A 6 × His Tag was introduced at the 5′ end of both the F and H gene fragments. The PA gene, which had a linker sequence introduced at the 5′ end, was amplified with primers listed in Table 1 by PCR using recombinant plasmid pFastBac1-eGFP-PA as a template (33). Subsequently, the fusion genes F-PA and H-PA were obtained through overlap extension PCR and then inserted into the pFastBac 1 vector (which already incorporated a GP64 signal sequence) via the *Xba* I and *Xho* I sites. The arrangement included the ectodomain of glycoprotein gene, a linker, and PA from the N-terminus to the C-terminus (Fig. 1). Recombinant bacmids, named rBacmid-F-PA and rBacmid-H-PA, were generated and then transfected into sf9 insect cells using Cellfectin II Reagent following the Bac-to-Bac expression system manual (Invitrogen, USA). Supernatants containing recombinant baculovirus rBV-F-PA and rBV-H-PA were harvested 5 days after transfection.

**TABLE 1:** Oligonucleotide primers in this study^*a*^

Primers	Sequences (5′−3′)	Restriction enzyme site
CDV-F-F	GCTCTAGAATG*CATCACCATCACCATCAC*CATCAATCCAACCTCAATGC	*Xba* I
CDV-F-R	**ACCAGAACCACCACCAGAACCACC**ACTGCCAAAATTAAAGGAAGAGCGC	
CDV-H-F	GCTCTAGAATG*CATCACCATCACCATCAC*CGATTTCACCAAGTATCAAC	*Xba* I
CDV-H-R	**ACCAGAACCACCACCAGAACCACC**ACGGTTACATGAGAATCT	
LinkerPA-F	**GGTGGTTCTGGTGGTGGTTCTGGT**GGTGCTTCTTCAGCTGGT	
LinkerPA-R	GCCTCGAGTTACTTGATACGCAGGTATTGACC	*Xho* I

^*a*^ Restriction enzyme sites are underlined. 6 × His Tag sequence is italicized. Central linker (Gly-Gly-Ser-Gly) ×2 base sequences are in bold.

### Preparation of BLPs displaying the F or H antigen of CDV

BLPs were prepared as described in detail elsewhere (57). In brief, the *L. lactis* strain MG1363 was harvested and resuspended in 10% trichloroacetic acid at a 0.2 culture volume. After boiling for 30 min, the hot acid-treated *L. lactis* was thoroughly washed with PBS to remove degraded DNA and proteins, resulting in the formation of BLPs. Using a Bürker Turk counting chamber, 2.5 × 10^9^ BLPs were defined as one unit (U).

To prepare the BLPs displaying the F or H antigen of CDV, 8 mL of the supernatants from suspension culture of sf9 cells infected with the rBV-F-PA or rBV-H-PA was collected and then mixed with 1 U of naked BLPs on a rotary shaker for 30 min at room temperature, resulting in the formation of BLPs-F and BLPs-H.

### Transmission electron microscopy analysis

The samples, including BLPs-F, BLPs-H, BLPs, and *L. lactis* MG1363, were fixed at 4°C overnight with 2.5% glutaraldehyde, respectively. Subsequently, ultrathin sections (70 nm) were sliced and stained with 2% uranyl acetate and Reynolds' lead citrate. Finally, the specimens were examined using a transmission electron microscope (H-7650, Hitachi, Japan).

### Identification of BLP-binding fusion protein

For indirect IFA, 100 µL of BLPs-F or BLPs-HN particles were resuspended in 3% bovine serum albumin (BSA) and blocked at 37°C for 30 min. Mouse anti His Tag monoclonal antibody (Beyotime Biotechnology, Shanghai, China) was used as the primary antibody and 1:500 diluted fluorescein isothiocyanate (FITC)-labeled goat anti-mouse IgG was used as the secondary antibody. After the final wash, the particles were observed with a Zeiss microscope and the Zeiss Axiovision digital imaging system (Zeiss, Oberkochen, Germany).

For western blot analysis, the samples of BLPs-F and BLPs-H were separated by 10% SDS-PAGE under denaturing conditions and transferred onto polyvinylidene fluoride membranes to identify BLPs binding the recombinant proteins. The samples of sf9 cells infected with rBV-F-PA or rBV-H-PA served as the control. Immunoblot analysis was performed using a monoclonal antibody against His Tag. Immunodetection was carried out using a horseradish peroxide (HRP)-conjugated goat anti-mouse IgG and enhanced chemiluminescence.

### Immunization studies

All animal experiments were carried out in accordance with the National Guidelines for Experimental Animal Welfare and approved by the Animal Care and Ethics Committees of Jilin Agriculture University (JLAU20221010005).

A mixture composed of equal amounts of BLPs-F and BLPs-H was prepared and designated as CDV-BLPs. For mouse immunization studies, a total of 45 4-week-old female BALB/c mice were randomly divided into three groups and intramuscularly vaccinated with 1 U of CDV-BLPs, with or without MnJ adjuvant, at a volume of 0.1 mL. The MnJ adjuvant was provided by MnStarter Biotechnology Co., Ltd. (Jiangsu, China). The third group of mice was inoculated with PBS as the negative control. Two booster immunizations with the same dose were administered at a 2-week interval. Blood samples were collected after 14, 28, and 35 days.

For dog immunization, 3-month-old beagle dogs (*n* = 3) without a history of CD vaccination and without virus-neutralizing antibodies against CDV were intramuscularly vaccinated with 4 U of CDV-BLPs with MnJ adjuvant or PBS at a volume of 1 mL. At 2 and 4 weeks after the initial vaccination, the dogs received second and third vaccine doses, respectively. Serum samples were collected at 14, 28, and 35 days post-immunization. On day 42 after the primary immunization, the dogs were intramuscularly challenged with the CDV TM-CC strain at a dose of 10^4^ TCID_50_ (58). Clinical signs were observed and recorded for 15 days. Rectal temperatures for all animals were recorded. A scoring system for other clinical sign-related CDV infection, including general condition, appetite, fecal consistency, ocular discharge, and nasal discharge (Table 2), was created and applied to quantitatively evaluate the response to challenge.

**TABLE 2:** Scoring system for clinical signs of canine distemper

Signs	Score value	Signs	Score value	Signs	Score value	Signs	Score value	Signs	Score value
General condition		Appetite		Fecal consistency		Ocular discharge		Nasal discharge	
Normal Mild Lethargy Neurological	0 1 2 3	Normal Good Bad Very bad	0 1 2 3	Normal Abnormal Watery Bloody	0 1 2 3	No Mild Moderate Severe	0 1 2 3	No Serous Mucous Mucopurulent	0 1 2 3

### Enzyme-linked immunosorbent assay

Mouse blood samples were collected to prepare serum at 14, 28, and 35 days post-immunization. The antibody end-point titer was measured using indirect enzyme-linked immunosorbent assay (ELISA). Briefly, 96-well plates were coated with purified CDV, obtained by sucrose density gradient centrifugation, at a concentration of 3 µg/mL in carbonate-bicarbonate buffer (pH 9.6) overnight. After washing with PBS, the 96-well plates were blocked with 5% BSA for 2 h at 37°C. Serum samples were diluted 1:200 in 100 µL of PBS and added to the plates for incubation. After an hour, the plates were washed with PBST buffer (PBS with 0.05% Tween 20) and incubated with HRP-conjugated goat anti-mouse IgG. Following washing, 100 µL of tetramethylbenzidine substrate was added to each well. The reaction was stopped with 2-M H_2_SO_4_, and the absorbance was read at 450 nm using an automated ELISA plate reader. For IgG subtype determination, IgG1 and IgG2a levels were determined by the same method as described for total IgG, except that HRP-conjugated anti-mouse IgG1 and IgG2a secondary antibodies were used, respectively.

Concentrations of IFN-γ and IL-6 in canine serum were determined using commercial ELISA test kits (both from R&D Systems, Minneapolis, MN, USA) following the respective manufacturer’s protocols

### Flow cytometry

On day 35 after the initial immunization, spleen lymphocytes from each group of mice were isolated. Single-cell suspensions were seeded in a 24-well plate at 1.5 × 10^6^ cells/well and incubated with purified CDV antigen (4 µg/mL) or medium only for 8 h. One microliter of BD GolgiPlug Protein Transport Inhibitor (containing Brefeldin A) was added and incubated for 4 h. Afterward, the cells were washed and stained with Alexa Fluor 700-conjugated monoclonal antibodies against mouse CD3 (clone: 500A2), PerCP-Cy5.5-conjugated antibodies for mouse CD4 (clone: RM4-5), and FITC-conjugated antibodies for mouse CD8a (clone: 53–6.7). The cells were fixed with the BD Cytofix/Cytoperm kit following the manufacturer’s protocol and then labeled intracellularly with PE-Cy7-conjugated anti-Mouse IFN-γ (clone: XMG1.2) or isotype control PE-Cy7-conjugated antibodies (BD Pharmingen) for 20 min at 4°C in the dark. After the final wash, the cells were analyzed with a flow cytometer (BD LSRFortessa, USA).

To detect changes in CD4^+^ and CD8^+^ T cell subsets in dogs, PBLs were isolated using the canine peripheral blood lymphocytes isolation kit (Beijing Solarbio Science & Technology Co., Ltd., China) 35 days after the initial immunization. Subsequently, 1 × 10^6^ PBL cells were stained with a dog T Lymphocyte Cocktail (anti-dog pan T cell-APC, anti-dog CD4-PE, anti-dog CD8-FITC) or an isotype control cocktail A obtained from BD Biosciences. Finally, the cells were analyzed using a flow cytometer (BD LSRFortessa, USA) and the FlowJo software (Tree Star, Ashland, OR).

### RT-qPCR assay

Nasal, throat, and rectal swabs were collected and vortexed in 1 mL of sterile PBS. After centrifuging at 10,000 rpm for 10 min at 4°C, the supernatant was used for viral RNA extraction using the TIANamp Virus DNA/RNA Kit (Tiangen, Beijing, China). Two microliters of RNA was subjected to an RT-qPCR assay targeting the CDV NP gene with the One Step PrimeScript RT-PCR Kit (Takara Co., Ltd., Dalian, China) on the ABI 7500 instrument, as described previously (59). The sequences of the primers and probe are the follows: N-forward, AGCTAGTTTCATCTTAACTATCAAATT; N-reverse, TTAAATCCCCAGAAAACTCATGC; N-prob, ACCCGAGAGCCGGATACATAGTTTCAATGC, which was labeled with the fluorescent reporter 6-carboxy-fluorescein at the 5′ end and the fluorescent quencher Black Hole Quencher 1 at the 3′ end. The reaction was performed as follows: 42°C for 5 min; 94°C for 10 s; then 40 cycles of 95°C for 5 s and 60°C for 30 s.

### Statistical analysis

All data are presented as the mean ± SD and were analyzed using either one-way analysis of variance or an unpaired Student’s *t*-test, as appropriate, with GraphPad Prism 9.2 software. A *P* value of less than 0.05 was considered significant for all comparisons. *P < 0.05; **P < 0.01; ***P < 0.001.

## Data Availability

The original contributions presented in the study are included in the article. Further inquiries can be directed to the corresponding author.
